# Neuroprotective Effects of Mitochondria-Targeted Plastoquinone and Thymoquinone in a Rat Model of Brain Ischemia/Reperfusion Injury

**DOI:** 10.3390/molecules200814487

**Published:** 2015-08-11

**Authors:** Denis N. Silachev, Egor Y. Plotnikov, Ljubava D. Zorova, Irina B. Pevzner, Natalia V. Sumbatyan, Galina A. Korshunova, Mikhail V. Gulyaev, Yury A. Pirogov, Vladimir P. Skulachev, Dmitry B. Zorov

**Affiliations:** 1 Belozersky Institute of Physico-Chemical Biology, Lomonosov Moscow State University, Leninskye Gory, House 1, Building 40, 119992 Moscow, Russia; E-Mails: silachevdn@genebee.msu.ru (D.N.S.); plotnikov@genebee.msu.ru (E.Y.P.); irinapevzner@mail.ru (I.B.P.); korsh@belozersky.msu.ru (G.A.K.); skulach@belozersky.msu.ru (V.P.S.); 2 Institute of Mitoengineering, Lomonosov Moscow State University, Leninskye Gory, House 1, Building 73A, 119992 Moscow, Russia; E-Mails: lju_2003@list.ru (L.D.Z.); sumbatyan@belozersky.msu.ru (N.V.S.); 3 International Laser Center, Lomonosov Moscow State University, Leninskye Gory, House 1, Building 62, 119992 Moscow, Russia; 4 Faculty of Chemistry, Lomonosov Moscow State University, Leninskye Gory, House 1, Building 3, 119992 Moscow, Russia; 5 Faculty of Fundamental Medicine, Lomonosov Moscow State University, Lomonosovsky Prospekt, House 31-5, 117192 Moscow, Russia; E-Mail: mihon-epsilon@yandex.ru; 6 Faculty of Physics, Lomonosov Moscow State University, Leninskye Gory, House 1, Building 2, 119992 Moscow, Russia; E-Mail: yupi937@gmail.com

**Keywords:** brain ischemia, mitochondria-targeted antioxidants, plastoquinone, thymoquinone, mitochondria

## Abstract

We explored the neuroprotective properties of natural plant-derived antioxidants plastoquinone and thymoquinone (2-demethylplastoquinone derivative) modified to be specifically accumulated in mitochondria. The modification was performed through chemical conjugation of the quinones with penetrating cations: Rhodamine 19 or tetraphenylphosphonium. Neuroprotective properties were evaluated in a model of middle cerebral artery occlusion. We demonstrate that the mitochondria-targeted compounds, introduced immediately after reperfusion, possess various neuroprotective potencies as judged by the lower brain damage and higher neurological status. Plastoquinone derivatives conjugated with rhodamine were the most efficient, and the least efficiency was shown by antioxidants conjugated with tetraphenylphosphonium. Antioxidants were administered intraperitoneally or intranasally with the latter demonstrating a high level of penetration into the brain tissue. The therapeutic effects of both ways of administration were similar. Long-term administration of antioxidants in low doses reduced the neurological deficit, but had no effect on the volume of brain damage. At present, cationic decylrhodamine derivatives of plastoquinone appear to be the most promising anti-ischemic mitochondria-targeted drugs of the quinone family. We suggest these antioxidants could be potentially used for a stroke treatment.

## 1. Introduction

Disorders of brain blood supply, particularly ischemic stroke, result in pathological reactive oxygen species (ROS) production by mitochondria, which is considered one of the key factors of neural cell damage and death. Oxidative stress, *i.e.*, the positive imbalance of ROS production over scavenging, is one of the main factors of organ dysfunction in various ischemic pathologies. It explains why cell survival and functional recovery depend on the prevention of ROS hyperproduction. Antioxidants are one of the basic drugs used to achieve this effect [1]. This group includes compounds of various chemistry and mechanisms of action, eventually affecting the free radical-induced oxidation of cellular structures and biomolecules (for example, membrane lipid peroxidation, DNA nucleotide oxidation, or protein carbonylation).

The most efficient antioxidants include various plant and animal compounds, which have been selected for millions of years of evolution possibly intended to maintain the cellular redox homeostasis. These compounds include vitamins A and E, ubiquinone, thymoquinone, and some other, usually plant-derived compounds, widely used in experimental and clinical studies. However, of note, ROS are also vitally important components responsible for intra- and intercellular signaling [2]. Thus, antioxidant treatment may change the ROS homeostasis of a cell and impair ROS-associated signaling. As a result, antioxidant treatment may lead to reductive stress and even paradoxical aggravation of oxidative stress [3,4,5]. It is likely antioxidants’ negative influence on signaling that explains the numerous failures of the clinical usage of antioxidants [6].

The solution to the problem of the negative side effects of antioxidants may be based on their targeted delivery to the needed destination on time, *i.e.*, to the damaged organ exactly upon oxidative stress. As it is known that mitochondria are one of the principle ROS sources in the cell under ischemia, the targeted delivery of antioxidant molecules to these organelles might enhance their antioxidant activity and in turn alleviate their effect on other ROS-signaling-related pathways of the cell.

This approach was implemented by Murphy’s group through synthesis of mitochondria-targeted ubiquinone and vitamin E, which have been shown to alleviate certain pathological conditions [7,8]. Plastoquinone, a component of plant photosystem II, might be another promising antioxidant for clinical use. A chloroplast generating oxygen is exposed to much higher oxidative challenge than a mitochondrion, which utilizes oxygen. Apparently, evolution resulted in the emergence of the electron transfer chain of the chloroplast of another quinone in addition to the ubiquinone involved in the mitochondrial respiratory chain of the same plant cell. In model systems, plastoquinone was shown to manifest better antioxidant properties than ubiquinone [9,10]. The usage of plastoquinone in the form of mitochondria-targeted compounds has been shown to enhance its antioxidative activity [11]. Another antioxidant with experimentally proven efficiency is the main component of *Nigella sativa* extract, thymoquinone, which if targeted by mitochondria might greatly increase its beneficial effect [12].

The aim of this study was to explore the neuroprotective properties of several plastoquinone or thymoquinone derivatives conjugated to various penetrating ions in an ischemic brain injury model.

## 2. Results

### 2.1. Mitochondria-Targeted Antioxidants Protect against Ischemic Brain Injury

**Figure 1:**
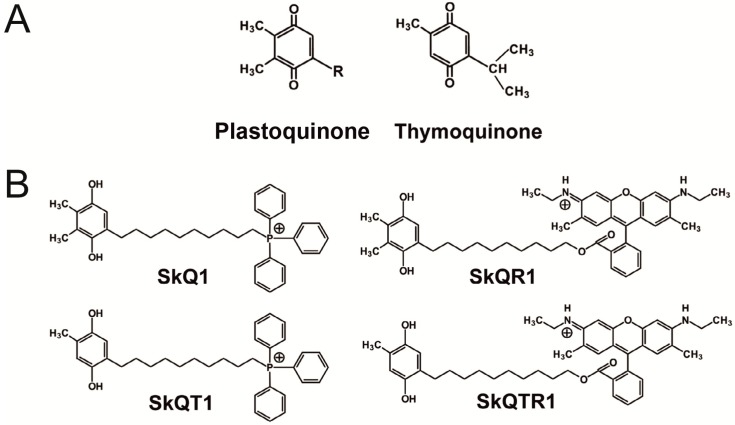
Chemical structure of compounds used in the study. (**A**) The compounds of plant origin, based on which mitochondria-targeted antioxidants were synthesized. R- nine isoprenyl units; (**B**) Chemical structures of: 10-(6′-plastoquinonyl) decyltriphenylphosphonium (SkQ1); 10-(6′-toluquinonyl) decyltriphenylphosphonium (SkQT1); 10-(6′-plastoquinonyl) decylrhodamine 19 (SkQR1); 10-(6′-toluquinonyl) decylrhodamine 19 (SkQTR1).

**Figure 2:**
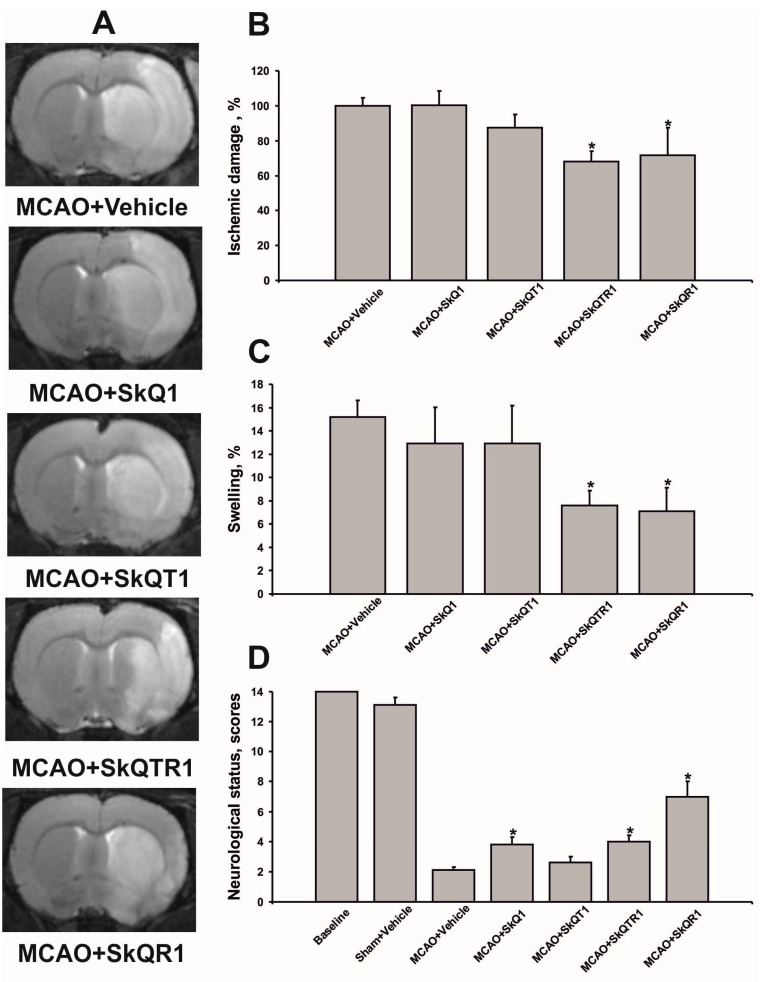
Post-insult mitochondria-targeted antioxidant treatment reduces ischemia/reperfusion-induced brain damage. Animals were subjected to ischemia for 1 h followed by reperfusion for 24 h. The mitochondria-targeted antioxidants were injected i/p immediately after the beginning of reperfusion at a dose of 1 µmol/kg. (**A**) Representative T2-weighted magnetic resonance (MR) images were obtained 24 h after reperfusion onset (each image covered an 0.8 mm thick brain section). Hyperintense regions in the right hemisphere (shown as more light area) refer to ischemic areas; (**B**) Infarct volume and (**C**) brain edema (swelling) evaluated by using magnetic resonance imaging (MRI) with analysis of T2-weighted images; (**D**) Neurological status estimated using limb-placing test. * denotes significant difference from the MCAO + Vehicle group (*p* < 0.05) (One-way ANOVA, followed by Tukey’s *post hoc* analysis for (**B**,**C**); Kruskal-Wallis test with the Mann-Whitney *u*-test for (**D**).

We examined the protective effects of various mitochondria-targeted plastoquinone and thymoquinone derivatives (Figure 1) on ischemic brain injury. Accordingly, using a model of the middle cerebral artery occlusion (MCAO), we tested the following compounds: 10-(6′-plastoquinonyl) decyltriphenylphosphonium (SkQ1); 10-(6′-toluquinonyl) decyltriphenylphosphonium (SkQT1) and 10-(6′-plastoquinonyl) decylrhodamine 19 (SkQR1); 10-(6′-toluquinonyl) decylrhodamine 19 (SkQTR1). Antioxidants were introduced intraperitonealy (i/p) immediately at the reperfusion onset at a dose of 1 μmol/kg. At 24 h after exposure of the rat brain to MCAO, we observed extensive cortical and striatal damage and also variable partial damage to the hypothalamus and amygdala outside the vascular territory of the middle cerebral artery. The treatment with SkQR1 and SkQTR1 significantly reduced the infarct volume to 72.0% ± 16.1% and 68.0% ± 6.1% compared to the vehicle-treated group, respectively (*p* < 0.05) (Figure 2A,B). Furthermore, ischemia caused significant swelling of the brain, occupying about 15.0% ± 1.4% of the volume of the hemisphere. Meanwhile, SkQR1 and SkQTR1 diminished brain swelling at least twofold to 7.1% ± 2.0% and 7.6% ± 1.6%, respectively (*p* < 0.05) (Figure 2A,C). The two other antioxidants, SkQ1 and SkQT1, had no significant effect on the volume of brain damage and swelling (Figure 2A–C). In addition, ischemia caused significant sensorimotor deficiency in the contralateral limbs relative to the damaged hemisphere. While the intact rats before the induction of ischemia scored 14.0 in a limb-placing test, and sham-operated animals scored 13.1 ± 0.5, rats after ischemia demonstrated only 2.1 ± 0.2. The treatment with SkQ1 and SkQTR1 restored the neurological status to 3.8 ± 0.5 and 4.0 ± 0.4 points, respectively (*p* < 0.05) (Figure 2D). SkQR1 was able to induce a robust improvement of the total score in the limb-placing test to 7.0 ± 1.0 points.

### 2.2. Dose-Dependent Neuroprotective Effects of SkQR1

**Figure 3:**
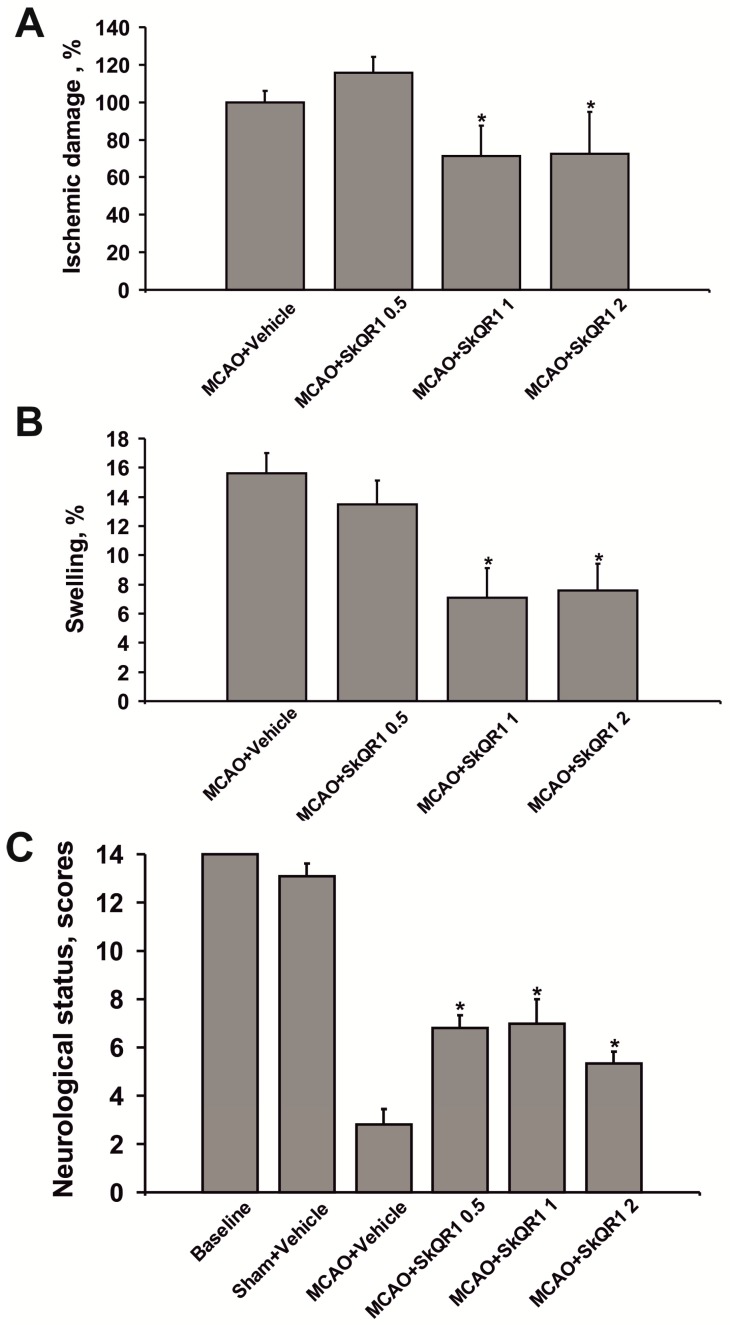
The treatment with SkQR1 protects the ischemia-injured brain. Rats were given i/p SkQR1 at doses 0.5, 1, or 2 μmol/kg after the beginning of reperfusion. (**A**) Infarct volume and (**B**) brain swelling measured in the MR T2-weighted images; (**C**) Neurological status estimated by limb-placing test. * denotes significant difference from the MCAO + Vehicle or MCAO groups (*p* < 0.05) (One-way ANOVA, followed by Tukey’s *post hoc* analysis for (**A**,**B**); Kruskal-Wallis test with the Mann-Whitney *u*-test for (**C**).

As SkQR1 proved to be the most efficient neuroprotector, we explored the dose-dependence of the compound’s neuroprotective activity. When compared with the vehicle group, the infarct volume was significantly reduced by 28% after treatment with 1 and 2 µmol/kg SkQR1 (*p* < 0.05) (Figure 3A). The treatment with 1 and 2 µmol/kg SkQR1 also significantly reduced brain swelling (*p* < 0.05) (Figure 3B). There were no statistically significant differences in infarct volume and swelling after treatment with 0.5 µmol/kg SkQR1. However, the treatment with SkQR1 at all doses (0.5, 1, and 2 µmol/kg) significantly improved functional recovery as measured by neurologic deficit scores (from 2.8 ± 0.7 to 6.8 ± 0.5, 7.0 ± 1.0, 5.3 ± 0.5, respectively) (Figure 3C).

### 2.3. Neuroprotective Effect of Long-Term Treatment by SkQR1

**Figure 4:**
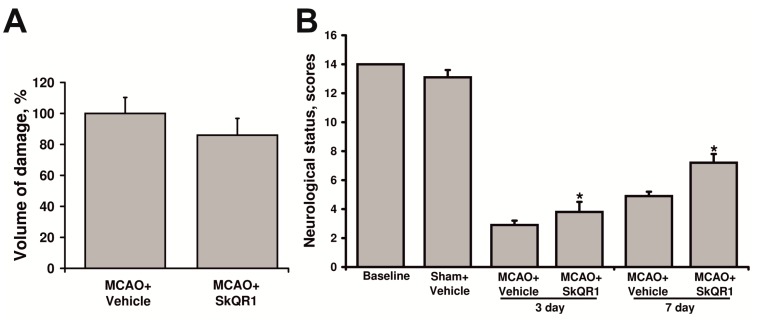
Neuroprotective effect of long-term treatment by SkQR1. SkQR1 at dose 0.1 μmol/kg was administrated i/p immediately after reperfusion and for a consecutive four days. (**A**) Infarct volume was measured in the MR T2-weighted images obtained seven days after MCAO; (**B**) Neurological status estimated using limb-placing test. Animals were tested on three and seven days after MCAO. * denotes significant difference from the MCAO + Vehicle *vs.* MCAO group treated with SkQR1 (*p* < 0.05) (the Mann-Whitney *u*-test).

In the next experiment, rats were treated with 0.1 µmol/kg SkQR1 i/p for four days. The first injection was made simultaneously with the reperfusion onset, and the other injections were made within a period of 24 h. Long-term treatment by SkQR1 had a trend of mitigating brain damage on day seven after MCAO (Figure 4A). The neurological status assessment showed spontaneous sensorimotor function recovery after ischemia even in untreated rats. However, SkQR1-treated rats demonstrated faster recovery of the sensorimotor functions of limbs starting from postoperative day three compared to the MCAO + Vehicle group. On day seven, the neurological score in SkQR1-treated rats exceeded the value for the MCAO + Vehicle group by 2 points.

### 2.4. Kinetics of SkQR1 Accumulation in the Brain

**Figure 5:**
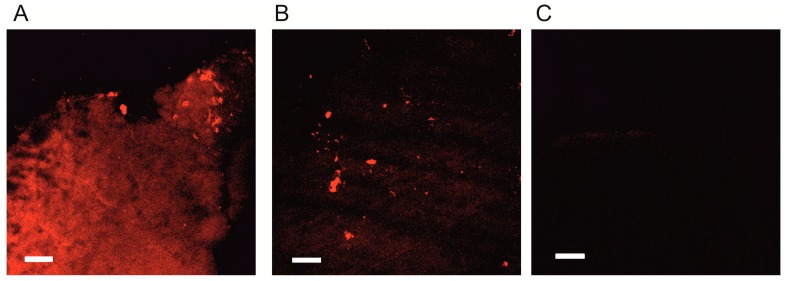
SkQR1 accumulation in the brain after intranasal administration. SkQR1 retention and distribution over brain compartments 1 h after i/n instillation of 1 µmol/kg SkQR1. Confocal microscopy of tissues slices. (**A**) The ipsilateral hemisphere and (**B**) the contralateral hemisphere; (**C**) As a negative control, brains from untreated animals were analyzed. Bar, 50 µm.

In order to explore the permeation of the blood-brain barrier (BBB) for SkQR1 after i/p or intranasal (i/n) injections, we analyzed its distribution in the brain using tissue sections sampled 1 h after different ways of administration at a dose of 1 µmol/kg. We observed that SkQR1 fluorescence in the brain was visible 1 h after i/n instillation. Remarkably, the fluorescence level in the ipsilateral hemisphere was higher than in the contralateral hemisphere, which indicates that SkQR1 directly penetrates into the brain when the i/n way of administration is used (Figure 5).

Instead, no global fluorescence of SkQR1 was observed after i/p injection (not shown) except for scattered bright fluorescent spots apparently attributed to the blood vessel cells. Apparently, for i/p injection, the BBB constrains penetration of SkQR1.

### 2.5. Neuroprotective Effect of Intranasal Administration of SkQR1

**Figure 6:**
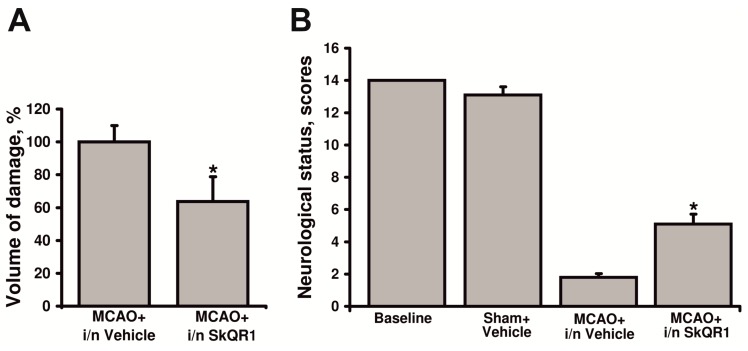
Neuroprotective effect of intranasal administration of SkQR1. (**A**) Infarct volume was measured in the MR T2-weighted images obtained 24 h after MCAO; (**B**) Neurological status estimated using limb-placing test. Animals were tested 24 h after MCAO. * denotes significant difference from the MCAO + i/n Vehicle *vs.* MCAO group i/n treated SkQR1 (*p* < 0.05) (Student’s *t*-test for (**A**); the Mann-Whitney *u*-test for (**B**)).

Our findings on the more effective SkQR1 penetration of the BBB after intranasal administration provided strong rationale to examine whether SkQR1 is able to protect brain tissue against ischemia-induced damage after such a method of administration. To address this question, we used instillation of SkQR1 into the nasal cavity at a dose of 1 µmol/kg or vehicle at the start of reperfusion. Analysis of the brain damage indicates that SkQR1 reduced infarct volume to 63.7% ± 15.0% *vs.* the vehicle-treated group (*p* < 0.05) (Figure 6A). To assess the beneficial effect of intranasal SkQR1 on neurological outcome, the rats were scored according to the severity of the neurological deficit. The results shown in Figure 6B demonstrate that rats treated with SkQR1 after MCAO had milder neurological deficits than animals from the MCAO + i/n Vehicle group.

## 3. Discussion

The study employed plant-derived mitochondria-targeted quinones such as plastoquinone or thymoquinone conjugated with penetrating ions decyltriphenylphosphonium or decylrhodamine 19 to obtain a lipophilic cation that easily penetrates through membranes. These antioxidant-carrying cations are specifically targeted to mitochondria, electrophoretically driven by electric potential formed by the mitochondrial respiratory chain. It was recently shown that these compounds are easily reduced by the respiratory chain, penetrate through model and natural membranes, specifically accumulate in mitochondria in an electrophoretic fashion, and strongly inhibit H_2_O_2_-induced apoptosis at pico- and nanomolar concentrations in cell cultures [11,12].

The electric locomotive principle concept of antioxidant delivery to the mitochondria was first suggested by Murphy and Smith [7]. They synthesized a compound composed of a thiobutyl residue chemically linked to a phosphonium cation. Later, thiobutyl was replaced by ubiquinone and this compound was named MitoQ [13]. Thereafter, MitoQ’s therapeutic potential was studied in various pathological models associated with oxidative stress [14,15,16]. However, MitoQ was administered predominantly before the induction of pathological conditions, which complicated the assessment of the drug’s clinical potential, since it was not clear whether it retained potency after being introduced in a period after the incidence of oxidative challenge (these conditions better reflect the clinical situation). Moreover, there are practically no experimental studies demonstrating MitoQ’s efficiency proven in brain pathologies. In the study by Hobbs *et al.*, a nulled positive effect of intracerebral MitoQ injection into the striatum was shown on a neonatal ischemia/hypoxia model [17].

Later, Skulachev’s group also synthesized mitochondria-targeted antioxidants containing positively charged phosphonium or rhodamine moieties tethered to plastoquinone by a decane linker [11]. Some of these mitochondria-targeted antioxidants demonstrated protective effects in oxidative stress-associated pathologies, such as ischemic brain, kidney, or bladder, rhabdomyolysis, or traumatic brain injury [18,19,20,21,22,23]. However, in most cases in these studies, the antioxidants were also administered before injury. In the present study, we explored the neuroprotective properties of mitochondria-targeted antioxidants administered after brain ischemia/reperfusion, analyzing different doses and ways of administration. Moreover, we compared the therapeutic efficiency of four mitochondria-targeted antioxidants differing in antioxidant moieties as well as penetrating ions. The conjugation with rhodamine residue has been chosen due to the simplicity of tracking the compound by fluorescence analysis, with a proven high permeability of rhodamines for membranes and their biological activity [24,25,26,27]. The highest neuroprotective efficiency was observed for antioxidants conjugated with rhodamine 19. SkQTR1 and SkQR1 equally diminished the damage area and the brain edema. However, the effect on neurologic deficiency was manifested more in SkQR1-treated animals when compared to other antioxidants. These data considered SkQR1 as a more efficient mitochondria-targeted antioxidant and it was chosen for further experiments.

The fluorescence-based study of SkQR1 penetration into the brain did not reveal SkQR1 fluorescence in the brain tissues when the drug was administered i/p. The confocal microscopy analysis of the brain sections revealed only small fluorescent regions, presumably blood vessels, which indicates that SkQR1 hardly passes through the BBB. However, intranasal administration into one nostril resulted in quite a strong fluorescence in the whole ipsilateral hemisphere, whereas in the contralateral hemisphere, fluorescence was very weak. These data suggest the possibility of direct delivery of SkQR1 into the brain, bypassing the BBB in case of intranasal administration. It is well known that drug instillation into the nasal cavity may ensure the targeted delivery of the drug into the brain through perivascular and perineural spaces [28]. Nevertheless, we detected no difference in SkQR1 neuroprotective effects between i/p and i/n administrations. Thus, SkQR1 neuroprotective activity does not correlate with its penetration into the brain, which may suggest that the effect is mediated by the protection of other tissues, for example, the endothelium. Brain ischemia is known to cause endothelial dysfunction [29], thus the protection of the endothelium may mitigate the nervous tissue damage [30].

Interestingly, we revealed differences in the neuroprotective effects of antioxidants conjugated with triphenylphosphonium and rhodamine 19. Ostensibly, that may be explained by the better penetration capacity of the molecules carrying rhodamine 19 as a hydrophobic cation, as it has been shown in various models [31]. However, we can not exclude some specific protective effects of the rhodamine residues.

The beneficial effect of mitochondria-targeted antioxidants that are lipophilic by their chemistry is greatly determined by the lipophilicity of oxygen itself and the majority of ROS (e.g., superoxide). Also, their positive effects may depend on parameters such as oxygen radical scavenging, penetration capability, and mitochondrial accumulation level and rate. Hydrophilic oxidants such as H_2_O_2_ are better scavenged by hydrophilic antioxidants (e.g., *N*-acetyl cysteine), while mitochondria-targeted antioxidants are less effective in scavenging H_2_O_2_ [32,33]. This premise dictates the necessity of thoroughly testing the whole range of such compounds for their neuroprotective efficiency, which may significantly vary depending on the nature of the antioxidant component and/or of the penetrating cation. In our study, we used plant quinones plastoquinone and thymoquinone as antioxidant components. Thymoquinone (2-demethylplastoquinone derivative) is the basic active component of black cumin (*Nigella sativa*) extract [34], which has demonstrated antioxidant properties in a number of studies [35,36]. Modern pharmacological studies have demonstrated that crude extracts of *Nigella sativa* seeds and purified thymoquinone might have neuroprotective effects against amyloid-β peptide-induced neurotoxicity in rat primary neurons [37]; 6-hydroxydopamine-induced neurotoxicity [38]; subarachnoid hemorrhage in rats [39]; global cerebral ischemia-reperfusion injury in rat hippocampi [40,41]; the kainate model of temporal lobe epilepsy [42] or 1-methyl-4-phenylpyridinium (MPP^+^); and rotenone toxicities of primary dopaminergic cultures [43].

Plastoquinone shares most chemical properties with thymoquinone and is present in all plant cells, being a photosystem II component. Plastoquinone was suggested to carry an antioxidative potency higher than ubiquinone since it occupies the compartment (chloroplast) exposed to an aggressive light-induced oxidative challenge due to the formation of molecular oxygen and to related reactive oxygen species [9,10]. However, to date, its antioxidant activity in various pathologies has scarcely been described in literature.

We may assume that the targeted delivery of thymoquinone and plastoquinone to the mitochondria might significantly increase the drugs’ antioxidant efficiency. Although mitochondria-targeted plastoquinone has been studied for several years [44], we are the first to compare its anti-stroke activity to that of mitochondria-targeted thymoquinone administered after ischemia.

Our study shows that in terms of the integral assessment of neuroprotective activity (the volume of the ischemic zone, brain edema, neurological deficiency alleviation), decylrhodamine-conjugated plastoquinone demonstrates the highest brain protection efficiency against ischemia. We conclude that therapeutic efficiency selection is needed for all antioxidants, including those targeted at mitochondria. We have shown recently that SkQR1 protection was mediated by its direct antioxidant effect, as well as by induction of signaling pathways stimulating the cell’s antioxidant protection, and, in particular, by inducing the synthesis of erythropoietin, a universal cytoprotector [20], and increasing GSK-3 phosphorylation, which is also a pro-survival signal [45]. This proves the possibility of some antioxidant-mediated beneficial antioxidant activity, which may be successfully used.

## 4. Experimental Section 

### 4.1. Use of Animals

The animal protocols used in this work were evaluated and approved by the institutional animal ethics committee (Protocol 2010_36). They are in accordance with the Federation of Laboratory Animal Science Associations (FELASA) guidelines. The experiments were performed on outbred white male rats (320–350 g). The animals had unlimited access to food and water and were kept in cages with a temperature-controlled environment (20 ± 1 °C) with light on from 9 a.m. to 9 p.m. For all surgical procedures rats are anesthetized with i/p injections of 300 mg/kg (12%) chloral hydrate. A feedback-controlled heating pad maintained the core temperature (37.0 ± 0.5 °C) during ischemia, supplemented with an infrared lamp until awake.

### 4.2. Chemicals

The chemical structure of compounds used in this study and the protocol of their synthesis are shown in Figure 1. The conjugates were synthesized by the Institute of Mitoengineering Lomonosov Moscow State University as previously described [11,12].

### 4.3. Middle Cerebral Artery Occlusion Model of Focal Ischemia

MCAO procedure and the sham operation were performed as previously described [46]. Briefly, rats were anesthetized with i/p injections of 300 mg/kg chloral hydrate. The right common carotid artery was exposed through a midline cervical incision. A heparinized intraluminal silicon-coated monofilament (diameter, 0.25 mm) was introduced via the external carotid artery into the internal carotid artery to occlude the blood supply to the middle cerebral artery region. A feedback-controlled heating pad supplemented with an infrared lamp was used to maintain the core temperature (37.0 ± 0.5 °C) during ischemia. After 60 min of occlusion, the filament was gently pulled out and the external carotid artery was permanently closed by cauterization. In sham-operated rats, the right common carotid artery was exposed and the external carotid artery was electrocoagulated without introducing the filament into the internal carotid artery. Ischemically damaged volume for each group was normalized to the mean for the group MCAO + Vehicle.

### 4.4. Study Design and Drug Treatment

#### 4.4.1. Comparing Various Mitochondria-Targeted Quinone Derivatives

The aim was to choose the most efficient mitochondria-targeted antioxidant in a group containing SkQ1, SkQT1, SkQTR1, and SkQR1 injected i/p at dose of 1 μmol/kg immediately after the beginning of reperfusion. The rats were randomly divided into the following groups: (1) SHAM + Vehicle (*n* = 6), (2) MCAO + Vehicle (*n* = 7), (3) MCAO + SkQ1 (*n* = 6), (4) MCAO + SkQT1 (*n* = 6), (5) MCAO + SkQTR1 (*n* = 6), and (6) MCAO + SkQR1 1 (*n* = 6).

#### 4.4.2. SkQR1 Dose-Dependent Effect Analysis

We analyzed groups MCAO + SkQR1 0.5 and MCAO + SkQR1 2, which received SkQR1 at doses of 0.5 and 2 μmol/kg, respectively. Substances were i/p injected immediately after the beginning of reperfusion.

#### 4.4.3. Analysis of a Long-Term SkQR1 Treatment

In this set of experiments, we studied the neuroprotective activity of SkQR1 administered chronically at low doses. SkQR1 at a dose of 0.1 μmol/kg was i/p administered immediately after reperfusion and for the consecutive 4 days (1 injection per day), and the MCAO + SkQR1 0.1 group was formed.

#### 4.4.4. Intranasal Administration

The rats were treated by i/n administration of SkQR1 immediately after reperfusion. Nasal drops (saline (20 µL) containing SkQR1 at a dose 1 μmol/kg) was administered with a small pipette every 5 min into both sides of the nasal cavity with 5 µL each instillation (total time of administration = 20 min). Rats were randomly divided into the following groups: MCAO + i/n Vehicle (*n* = 8), MCAO + i/n SkQR1 (*n* = 7).

### 4.5. MRI Studies

Infarct volume was quantified by analyzing brain MRI images obtained 24 h after the MCAO as described previously [47]. Brain swelling was also measured in the MRI images and calculated using the formula: swelling (edema) = (volume of right hemisphere − volume of left hemisphere)/volume of left hemisphere. Twenty-four hours after brain ischemia/reperfusion the rats underwent MRI of the brain. Under chloral hydrate anesthesia, the animal’s position was fixed with a special device which also maintained the animal’s temperature. MRI scans were acquired on a 7T MRI scanner (Bruker BioSpec 70/30 USR, Ettlingen, Germany). The imaging protocol included a T2-weighted image sequence (time to repetition = 4500 ms; time to echo = 12 ms; slice thickness = 0.8 mm).

### 4.6. Limb-Placing Test

The modified version of the limb-placing test consisting of seven tasks was used to assess forelimb and hindlimb responses to tactile and proprioceptive stimulation [48]. The rats were habituated for handling and tested before operation and after the reperfusion for 24 h. For each task, the following scores were used: 2 points, normal response; 1 point, delayed and/or incomplete response; 0 points, no response. Over seven tasks, the mean score was evaluated.

### 4.7. Analysis of SkQR1 Accumulation in the Brain

Rats were injected i/p or i/n with 1 µmol/kg SkQR1 and brains were excised after 1 h, fixed in 4% formaldehyde with PBS and sliced using a VibroSlice microtome (World Precision Instruments, Sarasota, FL, USA) into 100 µm thick sections. Slices were imaged with a LSM510 inverted confocal microscope (Carl Zeiss Inc., Jena, Germany) with excitation at 543 nm and emission collected at 560–590 nm. As a negative control, organs from untreated animals were used.

### 4.8. Statistics

Statistical analyses were performed using STATISTICA 7.0 for Windows (StatSoft, Inc., Tulsa, OK, USA). All data are presented as means ± standard error of means (SEM). Variance homogeneity was assessed with Levene’s test. Statistical differences between groups in the data of infarct volume and brain swelling were analyzed using one-way ANOVA with Tukey’s *post-hoc* test or Student’s *t*-test for independent samples. Statistical differences in the limb-placing tests between groups were analyzed using the Kruskal-Wallis test with the Mann-Whitney *u*-test (the Bonferroni *post-hoc* correction was applied) or Mann-Whitney *u*-test for independent samples. Values for *p* < 0.05 were assumed to be statistically significant.
